# Effect of an vitamin D deficiency on depressive symptoms in child and adolescent psychiatric patients – a randomized controlled trial: study protocol

**DOI:** 10.1186/s12888-018-1637-7

**Published:** 2018-03-01

**Authors:** Manuel Föcker, Jochen Antel, Corinna Grasemann, Dagmar Führer, Nina Timmesfeld, Dana Öztürk, Triinu Peters, Anke Hinney, Johannes Hebebrand, Lars Libuda

**Affiliations:** 1 Department of Child and Adolescent Psychiatry, University Hospital Essen, University of Duisburg-Essen, Wickenburgstr. 21, D-45147 Essen, Germany; 2 Pediatric Endocrinology and Diabetology, Kinderklinik II, University Hospital Essen, University of Duisburg-Essen, Essen, Germany; 3 Department of Endocrinology and Metabolism, Medical Center and Central Laboratory, University Hospital Essen, University of Duisburg-Essen, Essen, Germany; 4 Institute for Medical Biometry und Epidemiology, Philipps-University Marburg, Marburg, Germany

**Keywords:** Vitamin D, Depression, Mental health, Children & Adolescents

## Abstract

**Background:**

Depression is a significant health and economic burden worldwide affecting not only adults but also children and adolescents. Current treatment options for this group are scarce and show moderate effect sizes. There is emerging evidence that dietary patterns and specific nutritional components might play a role in the risk for developing depression.

This study protocol focusses on the role of vitamin D which is for long known to be relevant for calcium and phosphorous homeostasis and bone health but might also impact on mental health. However, the assessment of the vitamin D status of depressed juvenile patients, or supplementation of vitamin D is currently not part of routine treatment. Controlled intervention studies are indispensable to prove whether a vitamin D deficiency ameliorates depressive symptoms.

**Methods/design:**

This double blinded, randomized controlled trial will enroll 200 inpatients from a child and adolescent psychiatric department with a vitamin D deficiency defined by a 25(OH)-vitamin D-level < 30 nmol/l (12 ng/ml) and a Beck Depressions Inventory (BDI-II) score > 13 (indicating at least: mild depression). Upon referral, all patients will be screened, checked for inclusion criteria, and those eligible will be randomized after written consent into a supplementation or placebo group. Both study-arms will receive treatment-as-usual for their psychiatric disorder according to established clinical guidelines. The participants of the vitamin D supplementation group will receive 2640 I.E. vitamin D3 q.d. for 28 days in accordance with best practice in pediatric endocrinology. We hypothesize that delaying supplementation of vitamin D in the placebo arm will affect the treatment success of the depressive symptomatology in comparison to the vitamin D supplementation group. Patients will be enrolled for a period of 28 days based on the mean length of hospitalization of juveniles with depression.

**Discussion:**

Randomized controlled trials in children and adolescents with depression are needed to elucidate the role of a vitamin D deficiency for mental disorders and to investigate the relevance of a routine assessment and supplementation of vitamin D deficits.

**Trial registration:**

DRKS00009758, 16/06/2016 (retrospectively registered).

## Background

Depression is worldwide a leading cause of health and economic burden [1]. Efforts to improve depression outcomes substantially have failed thus far [2, 3], suggesting that factors not considered yet might influence risk, pathogenesis and persistence of depressive disorders. Consequently investments in additional options to current multimodal therapies are needed [4, 5]. Accumulating evidence suggests that lifestyle factors such as diet quality [6], but also physical activity [7] might contribute as risk or risk-mitigation factors, respectively to mental health issues in general [8] and depression specifically [9, 10]. Apart from a potentially promising adaptation to an overall healthier diet [11, 12] and life-style, some nutritional components [13] such as vitamin D might play an important role for mental health [14].

The term “vitamin D” subsumes several structurally related pleiotropic secosteriod hormones, which are usually not further sub-classified [15]. Vitamin D plays a dual role as hormone and fat-soluble vitamin, regulating the expression of more than 900 genes [16, 17] via binding to the vitamin D receptor (VDR) a steroid hormone receptor [18]. Vitamin D plays a key role in calcium and phosphorus homeostasis [15], bone health [19] and various cellular and neuromuscular functions [15]. The vitamin D receptor and vitamin D metabolizing enzymes are expressed in the brain [20, 21]. Due to its pleiotropic function vitamin D is also involved in signaling cascades and neurobiological pathways [20], which may affect mental health. The active metabolite 1,25(OH)_2_D_3_ is thought to modulate the differentiation and maturation of dopaminergic neurons [22] and to affect brain serotonin concentrations [20, 23]. Low vitamin D status is associated with a range of adverse neuropsychiatric outcomes [24–32]. In particular, population based epidemiological and clinical studies showed an association of low 25(OH)-vitamin D serum levels (25(OH)D) with depressed mood [14, 26, 33]. Thus “sub-optimal 25(OH)D levels” may precipitate mental disorders [34, 35] and achievement/restoration of “optimal levels” may foster mental health and offer potentially a treatment option. However, the definition of the “optimal-level” of 25(OH)D, is still under discussion [36] since all currently pursued cut-offs are related to bone health outcomes [37] and based on suppression of PTH levels [38] with no evidence available with respect to an “optimal-level” for mental health [20]. The Institute of Medicine (IoM) defines 25(OH)D levels > 50 nmol/l (equals 20 ng/ml) as sufficient, whereas levels of 30–50 nmol/l or < 30 nmol/l are classified as at risk for inadequacy or as at risk for deficiency [37, 39]. In general many cofactors influence the vitamin D status [14, 40]. Apart from seasonal [41] and other environmental factors the risk of vitamin D insufficiency is also influenced by genetic factors. Two genome-wide association studies (GWAS) of 25-hydroxyvitamin D levels revealed genome wide associations for Single Nucleotide Polymorphisms (SNPs) at five gene loci (*GC*, *DHCR7/NADSYN1*, *CYP2R1*, *CYP2R2*, *CYP24A1*) [42, 43] and a more recent GWAS in a Punjabi Sikh population replicated three out of the five known loci and discovered a new one between *FOXA2* and *SSTR4* [44].

Results from observational studies suggest a subtle but beneficial role of vitamin D in several mental health conditions in childhood and adolescence. Most studies including children and adolescents resort to Attention Deficit Hyperactivity Disorder (ADHD) or Autism Spectrum Disorders (ASD). However, randomized controlled trials are lacking [14]. In adults 21 RCTs could be identified by a literature search based on adults with depression (for details see [14]). Although the results from these studies are not directly transferable to childhood or adolescence, the overall findings as well as common limitations in the study designs of the studies in adults should be considered for future studies on depression in children and adolescents. Some common limitations of the identified studies in adults were remarkable: baseline 25(OH)D levels were not reported in some studies, not all studies included a placebo group and/or psychiatric patients with depressive symptoms. Moreover study populations, vitamin D supplementation doses and lengths of intervention were quite different (for details see [14]).

Most of the reported moderate to strong inverse relationships between 25(OH)D levels and several somatic diseases (cardiovascular diseases, diabetes, etc.) from prospective cohort studies in adults were only poorly supported (little to no effects) by interventional vitamin D supplementation studies [45]. These missing effects in randomized controlled trials could be due to reverse causality, i.e. that low vitamin D levels are not the cause, but rather a consequence of a general ill health status and inflammatory processes which -in turn- play a role in the occurrence of the examined diseases [45–47]. This hypothesis is currently vividly debated [48–50]. It is currently also discussed whether it might be impossible to correct pathologic processes through vitamin D supplementation in adulthood which were potentially caused by vitamin D deficiency in early life [50]. Considering the rapid brain development during childhood, which extends into later stages of adolescence [51], we hypothesize that in the context of vitamin D a focus on children and adolescents is especially meaningful with respect to potential therapeutic effects on mental disorders.

This article presents the study protocol for the trial: “Effect of an untreated vitamin D deficiency on depressive symptoms in child and adolescent psychiatric patients – a randomized controlled trial” [German Clinical Trial Registry Code: DRKS00009758]. This is a randomized, placebo controlled trial aiming to investigate the effect of a vitamin D deficiency on an inpatient psychiatric treatment of depressive symptoms in children and adolescents.

We hypothesize that psychiatric treatment in patients, in whom vitamin D deficiency is corrected without time-delay (treatment group) is more effective to reduce depressive symptoms, than in patients with untreated vitamin D deficiency.

## Methods

### Study design

The study is set up as a 28 days (based on the mean length of hospitalization of juveniles with depression), parallel group, double-blind, randomized, placebo-controlled trial to investigate the effect of an untreated vitamin D deficiency (placebo arm) on depressive symptoms in child and adolescent psychiatric patients.

We are currently enrolling 200 participants at one study site, the Department of Child and Adolescent Psychiatry, University Hospital Essen, University of Duisburg-Essen, Essen, Germany. In total, recruitment, intervention, and database lock are anticipated to be finalized within a two years period after First-Patient-First-Visit (FPFV).

Patients with a vitamin D deficiency, who meet the inclusion criteria (see below) and consented to the study will be randomized to receive either 2640 I.E. vitamin D q.d. based on recommendations for treatment of vitamin D deficiency in patients with chronic diseases (REF) or placebo on top of treatment-as-usual (TAU). Participants will be assessed psychometrically prior to study start and after 28 days with the self-rated Beck-Depression-Index –II [52] (BDI-II; selected instrument for inclusion criteria and primary endpoint) and additional psychological instruments (Table 1).

**Table 1:** Scheduled measurements

Investigated item	Psychometric and sociometric questionaires/instruments	Executed /completed by	Time point(s)	Reference
Depression	BDI-II	Patient	Baseline, and end of study	[83]
Depression	DISYPS-II DES SBB	Patient	Baseline and end of study	[84]
Depression	DISYPS-II DES FBB	Parents	Baseline and end of study	[84]
Intelligence and mental development status	IQ,HAWIK, CFT, Son-R	Patient	Baseline	[85]
Structured Interview for clinical diagnosis	KIDDIE-SADS-PL	Clinical interviewer	Baseline	[66, 86]
Impulsivity	BIS	Patient	Baseline and end of study	[87]
Problem behaviors	CBCL	Parents	Baseline	[88, 89]
Problem behaviors	YSR	Patient	Baseline	[88]
Socioeconomic status	SES (KiGGS)	Parents	Baseline	[90]
Vitamin D status related questionnaire	SUN EXP QUEST	Patient	Baseline	[91, 92]
Habitual food and nutrient intake	FFQ	Patient	Baseline	[54]
Physical Activity	IPAQ	Patient	Baseline	[62]

*Abbreviations BIS* Barrett Impulsivity Scale, *CBCL* Child Behavior Checklist, *YSR* Youth Self Report, *TRF* Teacher’s Report Form, *CFT* Culture Fair Intelligence Test, *KIDDIE-SADS-PL* Kiddie Schedule for Affective Disorders and Schizophrenia for School Aged Children Present Lifetime version (Semi-structured diagnostic interview), *DISYPS-II* Diagnostic System for Mental Disorders in Childhood and Adolescence (Questionnaire), *FFQ* Food Frequency Questionaire, *HAWIK* Hamburger Wechsler Intelligence Test for Children, *IPAQ* International Physical Activity Questionnaire, *SON-R* Snijders-Oomen Non-verbal Intelligence Tests, *SES* Socioeconomic status module from the  KIGGS nation-wide German Health Interview and Examination Survey for Children and Adolescents (KiGGS), *SUN EXP QUEST* Sun Exposure Questionnaire

At the end of the study (after 28 days) all patients (placebo and verum) are recommended to substitute 1000 I.E. vitamin D per day - in case of persisting deficiency - for the following eleven months.

### Study aims

Within this two-armed, double-blind, randomized controlled trial we aim to test the hypothesis that moderate to severely depressed children and adolescents (BDI-II sum score at admission > 13) with a vitamin D deficiency (25(OH)-D3 level < 30 nmol/l [equivalent to < 12 pg/ml]) who will be treated as usual, but will not be supplemented with vitamin D (*placebo* arm) for a period of at maximum 28 days, will end-up with a significantly higher BDI-II sum score, than those immediately supplemented with 2640 I.U. vitamin D3 q.d. (*verum* arm). The assessment of the vitamin D status is not part of current routine diagnostic in clinical psychiatric departments or psychiatric offices. This study is to the best of our knowledge the first randomized controlled trial (RCT) investigating the effect of watchful waiting with regard to vitamin D supplementation in children and adolescent inpatients with an elevated depression score. The primary outcome parameter is the BDI-II sum score. Secondary outcome parameters encompass the 25(OH)-D levels from baseline to study end as well as impulsiveness and depressive symptoms assessed with parent- and self-rating instruments.

### Participant eligibility

#### Inclusion criteria

Eligible participants will be recruited from the clinical sample seeking elective treatment as inpatients or in the clinical day-care unit of the Department of Child and Adolescent Psychiatry, University Hospital Essen, University of Duisburg-Essen, Essen, Germany. Eligible participants need to score above 13 in the self-rated Beck-Depression-Index–II (BDI-II), which is the threshold for a mild depression. Patients additionally must have a 25(OH)-Vitamin D3 blood serum level < 30 nmol/l (equivalent to 25(OH)-Vitamin D3 < 12 pg/ml).

The eligibility criteria include participants of both genders aged from 11.0 to 18.9 years and capable of providing written informed consent. For participants below 18 years of age, written informed consent must also be provided by parents or assigned foster parents, respectively. All patients are informed that participation is totally voluntary.

#### Exclusion criteria

Exclusion criteria include: a concurrent diagnosis of a severe somatic disease, (ascertained by the assessment of the medical history and medical examination at inpatient admission), renal disease, hypocalcemia and/or a blood plasma parathyroid hormone (PTH) level > 130 ng/ml, mentaI retardation (IQ < 70). Patients, who present with vitamin D deficiency and established hypocalcemia or PTH level > 130 ng/ml in the recruitment phase, will be excluded, referred to pediatric endocrinology for further assessment and treatment, including initiation of immediate treatment with vitamin D3.

#### Sample recruitment

**Fig. 1:**
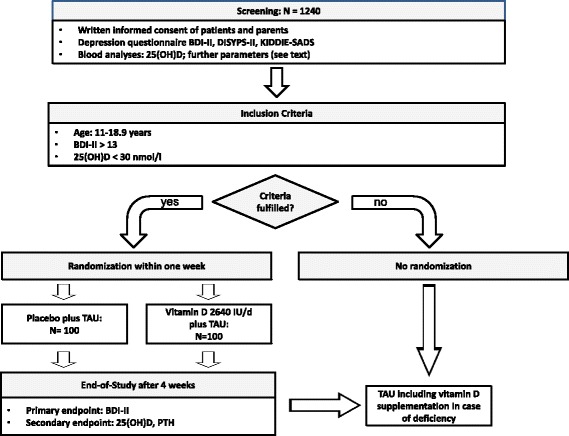
Study Flow-Chart. Abbreviations: 25(OH)D = 25(OH)-Vitamin D-level; BDI-II = Beck Depression Inventory II; KIDDIE-SADS-PL = Kiddie Schedule for Affective Disorders and Schizophrenia for School Aged Children Present Lifetime version (Semi-structured diagnostic interview); DISYPS-II = Diagnostic System for Mental Disorders in Childhood and Adolescence (Questionnaire); PTH = Parathyroid hormone; TAU = Treatment as Usual

Study participants will be recruited from the clinical sample eligible for elective inpatient or day-care treatment in the Department of Child and Adolescent Psychiatry, University Hospital Essen (see Fig. 1). Consent will be obtained by the admitting physician. Eligibility will be assessed within the first two days after hospital admission.

### Study procedure

The study is currently ongoing and scheduled for a duration of about two years and commenced with the “First-Patient-First-Visit” on June 15th 2016.

#### Screening assessment

Potentially eligible patients, who present to the University Duisburg-Essen Department of Child and Adolescent Psychiatry and who will be scheduled for hospital admission, will be informed about the ongoing study by a physician. If patients and parents are interested in an enrolment into the study and sign the written consent, patients will be asked to complete the BDI-II questionnaire [52] on the day of admission as the “primary endpoint intstrument” as well as the other psychological instruments as specified in Table 1. On the day of admission serum/plasma levels of 25(OH)D, calcium and parathyroid hormone will be determined in addition to the the clinical routine lab work.

Medical and psychiatric history will be obtained by the admitting physician to ensure eligibility. In this context the auxiologic parameters body height, weight, BMI and Tanner status [53] will be assessed and recorded as well as basic clinical parameters (e.g. blood pressure).

Potential participants and their parents will be asked to complete several psychometric and sociometric questionnaires, and for assessment of dietary intake a food frequency questionnaire (FFQ) [54], which was specifically designed to assess the food and nutrient intake with a particular focus on fatty acids of school-aged children in Germany and a questionnaire regarding several questions to explore factors influencing the vitamin D status as e.g. time spent outdoors and sun-shield use (see Table 1). In addition, the status of omega-3 long-chain fatty acids (n-3 LC-PUFA) in erythrocytes [55, 56], will be determined at baseline [57, 58]. First studies have shown reduced n-3 LC-PUFA levels in depressed adolescents in contrast to healthy matched controls [56].

#### Randomization and blinding

Children and adolescents meeting the above described eligibility criteria will be randomized as early as the third day, but at latest on the fifth day after admission for inpatient treatment. The study nurse under supervision of the principal investigator will transmit the following data to the Institute for Medical Biometry und Epidemiology (IMBE), Philipps-University Marburg, Marburg, Germany: pseudonymized patient code; BDI-II total score and 25(OH)D serum level. The IMBE will perform a block-wise randomization procedure and return a randomization code back to the study center which triggers the selection of the accordingly coded pill dispenser, which contains(blinded-mode) either vitamin D or identically looking placebo pills. The randomization is balanced for two BDI-II strata (total score values 14–23 and equal or greater than 24, respectively) and two 25(OH)D strata (25(OH)D < 12.5 nmol/l and 25(OH)D between 12.5–30 nmol/l). For 200 participants scheduled in total for randomization, the company Dr. B. Scheffler Nachf. GmbH & Co.KG (Bergisch Gladbach, Germany) has supplied 120 pill dispensers (100 for the protocol and 20 for losses/eventualities) with three times thirty vitamin D pills (dose strength: 880 I.E. per pill) and 120 dispensers with three times thirty identically looking placebos. These supplies will be sufficient for the planned 28 days of dosing with 2640 I.E. of vitamin D per day. The block-randomization code was generated prior to study start by the IMBE and sent to the company for an appropriate labeling of the pill dispensers.

### Study conditions

#### Vitamin D supplementation and placebo group

Vitamin D3 or placebo will be provided through pill dispensers to the patients (2640 I.U. as three pills). The pill dispensers labeled with the randomization code and containing either verum or placebo will be stored on the ward. The ward nurse will dispense the pills to the patients, matching the labeled dispenser with the patient’s randomization number every morning right after breakfast.

All participants will be treated for their respective disorders according to best clinical practice and existing treatment guidelines. There will be no separation or other special treatment of study participants as compared to other inpatients apart from the daily dispensing of study supplements and the recording of data within the CRF in addition to the standard patient records.

#### Data collection and outcome measures

Blood samples and a spot urine sample will be collected in the Department of Child and Adolescent Psychiatry as part of the clinical routine at admission. After written consent is obtained, the aliquots will be transferred at 4° within an hour after collection according to documented and approved standard operation procedures (SOPs) to the central laboratory of the University Hospital Essen. 25(OH)vitamin-D3, PTH and serum calcium levels will be measured and reported on the same day to a pediatric endocrinologist for approval. In addition electrolytes (including calcium), the differential blood count (DBC), thyroid-stimulating hormone level, inflammatory markers, liver and renal parameters, and alkaline phosphatase will be analyzed and reported.

Data for eligible participants will be transferred to the study nurse for further processing and inclusion into the CRFs. Remaining aliquots of blood samples and urine will be stored in at − 80 degree until further analyses. Planned assays are related to neutrophines, metabolites and hormones involved in metabolic signaling cascades. In case of consent from study participants and parents, DNA/RNA will be retrieved and stored for potential follow-up investigations of genetic variants and expression profiles of vitamin D metabolism related genes and gene products, respectively.

All applied psychometric instruments, sociometric questionnaires and outcome measures are displayed in Table 1. Depressive symptom severity is assessed as part of the screening by means of the BDI-II (self-rating) at the first visit to the clinic. At admission, clinical data including blood pressure and heart rate, use of medication and/or drugs (incl. Alcohol, smoking), puberty status according to Tanner [53], and auxiologic parameters (body height, body weight, and height and weight at birth) will be recorded. On the second day patients will meet with a therapist and the study nurse for a psychiatric interview and the completion of psychometric questionnaires. Patients will be asked to complete the BDI-II/ DISYPS-II -DES [52, 59] and the Barratt Impulsiveness Scale (BIS-11) [60]. Within the first week of hospitalization they will also be guided and asked to complete the CBCL (German version for school-aged children and adolescents of the Child Behavior Checklist [61]), Youth Self Report (YSR; German version [61] for patients and parents) and the International Physical Activity Questionnaire (IPAQ) [62]. The amount of sunlight exposure and outdoor activity will be assessed with a specialized questionnaire [63]. In order to study dietary habits and overall nutrition behavior as potential impact factors on mental health and co-variables for this study a Food Frequency Questionnaire FFQ [64] will be conducted.

Parents will be asked to answer questionnaires regarding their socioeconomic status (SES) [65].

The results of the initial clinical evaluation is then presented to a physician for children and adolescents psychiatry, who subsequently conducts a 30 to 60-min diagnostic evaluation according to DSM-IV criteria by means of the K-SADS-PL [66]. Within this session the medical history (psychiatric and somatic diagnoses) and previous treatment history will be obtained. Eligible patients will be randomized at the earliest and latest at day three and five after admission.

At the end of the study (at day 28 of the intervention) participants will complete the BDI-II/ DISYPS-II -DES [52, 59] and additional instruments as depicted in Table 1. A blood sample for the determination of 25(OH)D levels will be obtained. The same procedure will be applied – if possible and accepted by the participant – in case of premature study end (drop-out for any reason). In case of discharge from hospital before 28 days, participants will be asked to continue supplementation with the handed-out pill dispensers in the scheduled manner. These outpatient participants will be followed-up weekly by phone and asked to show up for the end-of-study examination at day 28 after study start.

A technical plausibility and completeness check of the data will be performed after data entry by a non-scientific assistant at the Institute for Medical Biometry und Epidemiology, Philipps-University Marburg (IMBE).

#### Data management

The ethical principles of the data handling and integrity were discussed with and approved by the Institutional Review Board (IRB). During the study a dedicated study nurse, supervised by the principal investigator, is responsible for completion and storage of the case record forms (CRFs). Hard copies of the CRFs are kept in a separate and secured filing cabinet within the department/study site. Data entry into the password protected study database is performed by the study nurse as well.

#### Study integrity

The study has been designed and will be conducted and reported in accordance with SPIRIT STATEMENT [67] and was registered (DRKS00009758) in “The German Clinical Trials Register” [68].

#### Sample size

We intend to recruit 200 participants. The sample size calculation was based on the study report by Mozaffari-Khosravi et al. [69] as the most comparable RCT. They applied the BDI-II as primary outcome measure in a population of adult depressed patients and observed a difference of four BDI-II score points between treatment arm (single dose vitamin D; 150 I.E.) and control group. Since the study was conducted in adults, we further looked for RCTs in adolescents with major depressive disorders, irrespective of vitamin D as the treatment item [70, 71], revealing a standard deviation of nine BDI-II score points as input for the sample size calculation, which yielded finally a sample size of 81 participants (two-sided t-test; alpha = 0.05; powered at 80% to detect a true difference). Attrition [72] was assumed to be 20%, demanding a recruitment of 100 participants per intervention arm.

#### Data analyses and statistical hypothesis

Analysis of the data will be conducted by a researcher blinded to the treatment condition. All statistical analyses will be performed after database-lock with “R” (www.r-project.org) at the Institute for Medical Biometry und Epidemiology (IMBE), Philipps-University Marburg, Marburg, Germany.

The main hypothesis of this study is, that treatment of depressive symptoms results in significantly lower improvement of BDI-II scores in the group of patients with vitamin D deficiency (placebo group) than in the supplementation group (verum group). The statistical hypothesis to be tested for the mean BDI-II score in the verum arm versus the placebo arm after 28 days of supplementation:

Null hypothesis: H_0_: BDI-II_verum_ = BDI-II_placebo_.

Alternative Hypothesis: H_1_: BDI-II_verum_ unequal BDI-II_placebo_.

A covariance analysis (ANCOVA) will be performed to contrast the outcome of the primary endpoint BDI-II in both intervention arms (two-sided level of significance; alpha = 0.05) in the Intention-To-Treat (ITT) population. The following co-variables will be considered: BDI-II at admission, 25(OH)D level at admission, age, and sex. Missing values will be considered via Last-Observation-Carried-Forward (LOCF) data, as far as available. Several sensitivity analyses will be conducted to prove for robustness, encompassing a per protocol analysis.

The secondary endpoint 25(OH)D and PTH levels will also be analyzed in the ITT population via an ANCOVA with the co-variables: 25(OH)D level at admission, age, and sex.

Further sensitivity analysis will be performed e.g. non-parametric analysis where patients with missing data will be set to lowest or highest ranks and repeated measure models will be applied. to hopefully confirm the results.

## Discussion

Depressive disorders in childhood and adolescents are devastating and may affect the whole lifespan [73]. Treatment options for this age group are scarce [74, 75] and show only low to medium effect sizes [76, 77]. The drug development pipeline for psychiatric disorders is rather dry and no breakthroughs – especially not for the young population – can be expected in due time [78]. Driven by the medical need and the search for benign, complementary options, lifestyle management strategies including physical activity, healthier diets [13, 11] and strategies for vitamin supplementation [79] appear on the scene and should be scientifically and clinically investigated according to best practices [79].

Based on findings from observational studies one promising option to improve depressive symptoms is a supplementation with vitamin D. However RCTs in young psychiatric patients have not been performed [14]. Several of the reported RCTs in adult depression revealed methodological issues and biological flaws as brought to attention by Spedding [80], who found positive results only in cases of baseline 25(OH)D levels below current cut-offs and clinically diagnosed mental disorders: In studies without biological flaws, a statistically significant improvement in depression with vitamin D supplements [SMD = 0.78; 95 CI (0.24, 1.27)] was found, whereas in studies with biological flaws a worsening in depressive symptoms [SMD = − 1.1; 95 CI (− 0.7, − 1.5)] was found (*p* values were not reported). Surprisingly, many studies neither considered a pronounced vitamin D deficiency nor a clearly defined disease status, which might have led to biased conclusions. Thus RCTs focusing on effects of vitamin D in well defined, homogenous groups [45–47] of children and adolescencets with mental disorders are of substantial importance to clarify the importance of the vitamin D status on mental health.

The current study aims to increment the evidence base. Since this study was set-up to prove the effect of untreated vitamin D deficiency within a clinical setting with potentially limited hospitalization time, the intervention duration of 28 days is seen as a potential limitation and risk for the study outcome. However, in case of success this clinical setting might have an even higher impact on treatment strategies for the sake of children and adolescents with depressive disorders.
